# NK cell adoptive transfer in acute myeloid leukemia: a systematic review and meta-analysis

**DOI:** 10.3389/fimmu.2026.1717413

**Published:** 2026-03-25

**Authors:** Letícia Dalla Vecchia Grassi, Gabriela de Toledo Passos Candelaria, Isabella de Oliveira Dias, Aline Raposo Nishimoto, Karina Fontão, Felipe Medauar Reis de Andrade Moreira, Heitor Duarte de Andrade, José Mauro Kutner, Nelson Hamerschlak, Juliana Aparecida Preto De Godoy, Leonardo Javier Arcuri, Lucila Nassif Kerbauy

**Affiliations:** 1 Hospital Israelita Albert Einstein, Division of Hemotherapy and Cell Therapy, São Paulo, Brazil; 2 Hospital Israelita Albert Einstein, Division of Hematology and Stem Cell Transplantation, São Paulo, Brazil; 3 Hospital Israelita Albert Einstein, Academic Research Organization, São Paulo, Brazil; 4 Hospital Israelita Albert Einstein, Faculdade Israelita de Ciências da Saúde, Instituto Israelita de Ensino e Pesquisa, São Paulo, Brazil

**Keywords:** acute myeloid leukemia, cellular immunotherapy, efficacy, natural killer cells, safety

## Abstract

**Background:**

Acute myeloid leukemia (AML) remains associated with high relapse rates and poor long-term survival, particularly in refractory patients or those ineligible for hematopoietic stem cell transplantation (HSCT). Adoptive transfer of natural killer (NK) cells has emerged as a promising immunotherapeutic strategy due to intrinsic cytotoxicity, low risk of graft-versus-host disease (GVHD), immune restoration capacity, and feasibility of allogeneic off-the-shelf manufacturing.

**Methods:**

A systematic review of phase 1–2 clinical trials evaluating non–genetically modified NK-cell adoptive transfer was performed using PubMed and EMBASE (2000–2024). Study selection and data extraction were conducted independently by two reviewers in Rayyan^®^. Study characteristics, interventions, and outcomes were extracted. Response rate in relapsed/refractory (R/R) AML receiving NK monotherapy was pooled using single-proportion meta-analysis. One-year disease-free survival (DFS) in other settings was pooled by inverse variance using logarithm-transformed rates. Heterogeneity (I²), publication bias, and study quality (NIH before–after tool) were assessed.

**Results:**

Of 790 records identified, 29 studies were included, predominantly single-arm phase I/II trials (one randomized phase II). In R/R AML (11 studies; 217 patients), pooled response rate was 35% (95% CI 29–42; I²=30%), with 20.2% proceeding to HSCT. Higher responses were observed in strategies enhancing NK activation or persistence, including expanded multi-infusion platforms and CIML-NK. In low-/intermediate-risk AML in remission (3 studies; 38 patients), pooled 1-year DFS was 82% (95% CI 56–100; I²=0). In high-risk AML in remission and ineligible for HSCT (5 studies; 59 patients), pooled 1-year DFS was 26% (95% CI 12–55; I²=84). In studies combining NK transfer with haploidentical HSCT (9 studies; 303 patients), pooled 1-year DFS was 40% (95% CI 27–57; I²=94). Toxicities were generally mild, GVHD rates were low (0–8%), and severe events were uncommon. NK-cell persistence was typically short, improving with multiple infusions and CIML approaches.

**Conclusion:**

Adoptive allogeneic NK-cell transfer appears safe and clinically promising, particularly for patients unfit for intensive therapy or HSCT. At present, its use should remain limited to clinical trials. Future studies should define the optimal approach that maximizes clinical activity while maintaining low toxicity and achieving durable persistence of allogeneic NK cells.

## 1 Introduction

Acute myeloid leukemia (AML) is a biologically heterogeneous malignancy characterized by clonal expansion of immature myeloid precursors and represents the most common acute leukemia in adults (1). Current treatment strategies range from intensive to low-intensity chemotherapy, with or without allogeneic hematopoietic stem cell transplantation (HSCT), and are guided by patient age, functional status, and risk, as defined by the European LeukemiaNet (ELN) classification (2). Despite recent advances, including targeted agents (FLT3 and IDH inhibitors), hypomethylating agents (HMA), and venetoclax-based regimens, long-term outcomes remain poor, with a 5-year overall survival of approximately 32%, underscoring the need for more effective therapeutic approaches (3, 4).

Cell-based immunotherapies have transformed the treatment of several hematologic malignancies, most notably through chimeric antigen receptor T-cell (CAR-T) therapies (5). However, in AML, CAR-T development has been limited by the lack of leukemia-specific antigens and the high risk of on-target toxicity against normal hematopoietic cells (6).

Given the limitations of CAR-T therapy in AML, alternative cell-based approaches, such as natural killer (NK) cell adoptive transfer, are under investigation. Physiologically, NK cells are key components of the innate immune system and play a central role in early immune surveillance against virally infected and tumor cells. Unlike T cells, NK cells do not require prior antigen sensitization. They can rapidly eliminate target cells through cytotoxic mechanisms or antibody-dependent cellular cytotoxicity, contributing to immune homeostasis and tumor immunosurveillance (7).

Studies have shown that NK cell counts are lower during active phases of AML (diagnosis and relapse) and increase during remission (8). Moreover, patients display an abnormal NK phenotype, with reduced activating receptors, increased inhibitory receptors, and impaired functionality, failing to eliminate blasts (9, 10). These observations support a direct role for NK cells in immune evasion mechanisms in AML. Accordingly, adoptive NK cell transfer has been explored as a strategy to provide functionally competent NK cells in the context of impaired endogenous NK cell activity.

**Figure 1:**
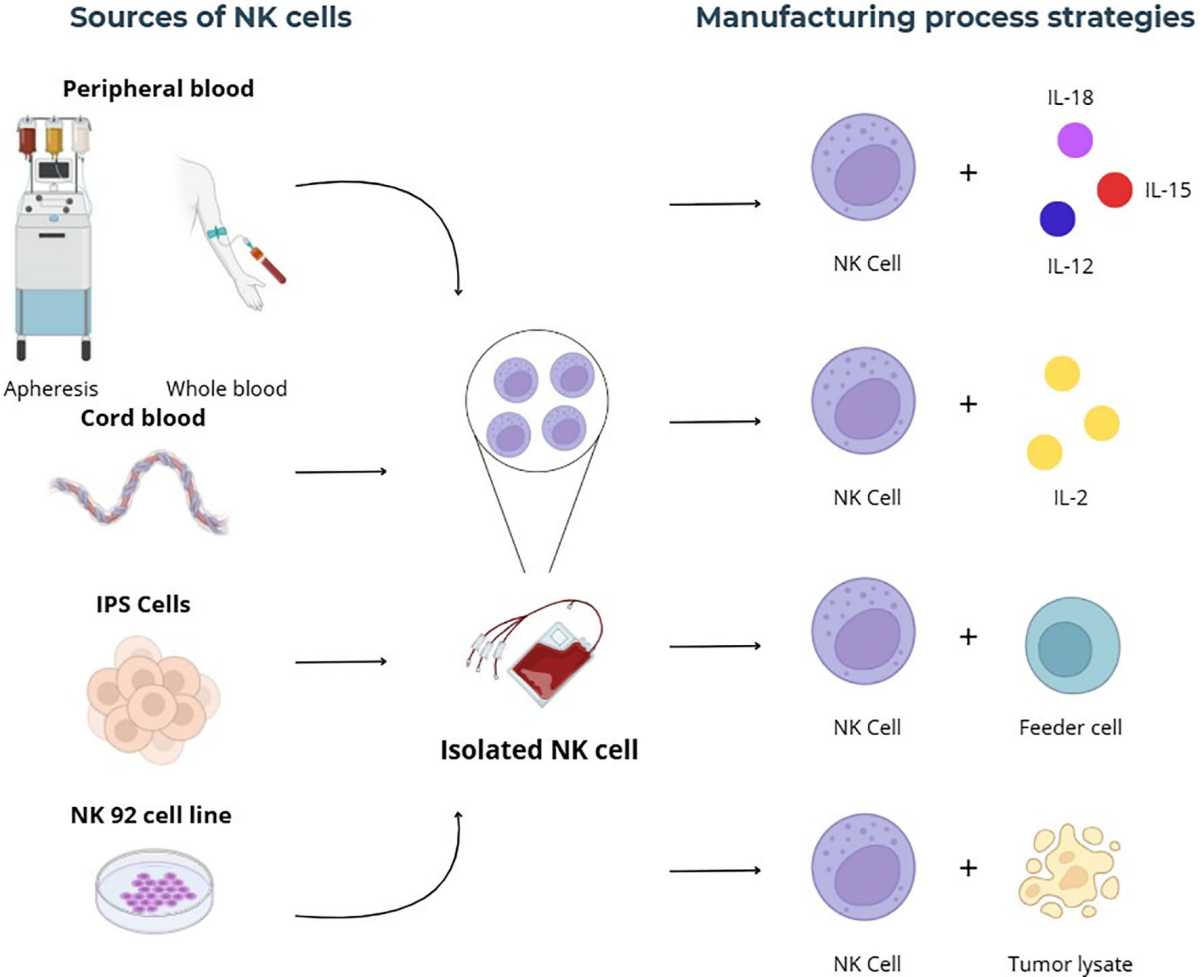
Sources and manufacturing strategies of NK cell therapies. IPS, induced pluripotent stem cells; IL, interleukin.

Several factors may influence the clinical performance of NK cell adoptive transfer, including cell source, expansion protocol, dose, associated chemotherapy, and the timing of the infusion. NK cells can be obtained from sources such as peripheral blood, umbilical cord blood, induced pluripotent stem cells (iPSCs), and tumor cell lines such as NK-92 (11, 12). The sources of NK cells used to manufacture this cellular therapy are depicted in **Figure 1**.

Advantages of NK cell adoptive transfer include the availability of allogeneic, off-the-shelf products and the feasibility of readily accessible cell banks. Additionally, compared to other allogeneic products such as CAR-T cells and HSCT, NK cells pose a lower risk of graft-versus-host disease (GVHD) (12).

Although NK cell adoptive transfer has emerged as a promising immunotherapeutic strategy for AML, the available evidence remains fragmented and heterogeneous. In particular, most published reviews aggregate genetically modified and unmodified NK cell platforms or focus primarily on clinical outcomes, providing limited insight into manufacturing-related variables that critically influence cell quality, feasibility, and reproducibility. Rapid advances in the field have generated new data that have not yet been systematically integrated. To address these gaps, we conducted a systematic review to comprehensively synthesize updated evidence on the use of non–genetically modified NK cells in AML, with a dedicated focus on manufacturing approaches, treatment settings, examining therapeutic strategies and clinically relevant outcomes, including safety, response, disease-free survival, and overall survival. By integrating clinical and manufacturing data, this work aims to provide a more refined and practical framework to inform future research and translational strategies in NK cell adoptive transfer for AML.

### 1.1 Objective

This study aims to conduct a systematic literature review of NK cell adoptive transfer in the treatment of AML. The objective is to evaluate current evidence on the efficacy, safety, and clinical applicability of this therapy and to identify treatment regimens and NK cell manufacturing methods employed in this context. Ultimately, this review seeks to clarify the role of NK cell adoptive transfer in AML and to highlight the main challenges in its development.

## 2 Methods

### 2.1 Eligibility criteria

We included clinical trials at any phase that evaluated AML patients treated with NK cell adoptive transfer therapy. No restrictions were applied regarding patient age or disease status. Only studies using non-genetically modified NK cells – isolated and expanded, with or without cytokines and feeder cells – were included. Eligible studies had to report clinical outcomes, such as response rate, relapse-free survival, and overall survival.

### 2.2 Search methods

Eligible studies were identified through searches in PubMed and EMBASE. The search was conducted in English on December 7, 2024, covering the period from 2000 to 2024.

The complete search strategy, including the MeSH terms used and the specific search strings applied in PubMed and EMBASE, is available in the **Supplementary Material**.

### 2.3 Study selection

The Rayyan^®^ platform was used for study selection and duplicate removal. Two authors independently screened titles and abstracts to identify potentially eligible studies for full-text review. Disagreements were resolved by a third reviewer. This exact process was also used to select the included studies and to extract the data.

### 2.4 Data extraction

The following data were extracted from the included studies:

- Study characteristics: author, title, year of publication, and clinical trial phase.
- Population characteristics: age, number of patients treated, disease stage, and prior treatments.
- Intervention details: number of NK cell infusions, NK cell source, autologous or allogeneic donor, NK cell manufacturing method, NK cell dose used, systemic interleukin use, lymphodepletion, rescue chemotherapy or conditioning.
- Clinical outcomes: response rate percentage of patients referred for HSCT, disease-free survival (DFS), overall survival (OS), incidence of GVHD, and other NK-related toxicities.

### 2.5 Analysis

The primary outcome for studies in relapsed or refractory patients and sole NK-therapy was response rate, which was summarized with a single-proportion meta-analysis. Heterogeneity was measured using I^2^, and we considered I^2^ values above 40% substantial heterogeneity, in which case we would use a random-effects model rather than a fixed-effects model. In other settings, the primary outcome was one-year disease-free survival. Disease-free survival rates were log-converted, and the standard error was estimated by the 95% confidence interval. A meta-analysis was carried out using the inverse-variance method. When the 95% confidence interval was not reported, we estimated the standard error by a linear function of the number of patients and the standard errors. Publication bias was assessed by visual inspection of the funnel plot and linear regression test of funnel plot asymmetry. The analysis was performed in R (R Foundation for Statistical Computing, Vienna, Austria, version 4.4.1) using the ‘meta’ package.

### 2.6 Quality assessment

Study quality was assessed using the NIH Quality Assessment Tool for Before-After (Pre-Post) Studies With No Control Group, given that the studies were phase 1 or 2 trials without randomization or a control group. The tool was independently applied by two reviewers, and in cases of disagreement, a third reviewer provided the final assessment.

## 3 Results

### 3.1 Study identification and selection

A total of 192 citations were retrieved from PubMed and 598 from EMBASE, resulting in 790 records. After removing 68 duplicates, 722 studies remained for abstract screening.

Following this step, 658 records were excluded, and 52 studies were selected for full-text review.

After full-text analysis, 23 studies were excluded for the following reasons: interim analyses or subgroup analyses of already included studies (n=8); no results reported (n=5); partial results considered insufficient, presented as posters at scientific conferences (n=4); non-evaluable results (n=3), in which the intervention or population could not be clearly identified; inadequate study design (n=2), including one preclinical study and one case report; and incorrect population (n=1).

**Figure 2:**
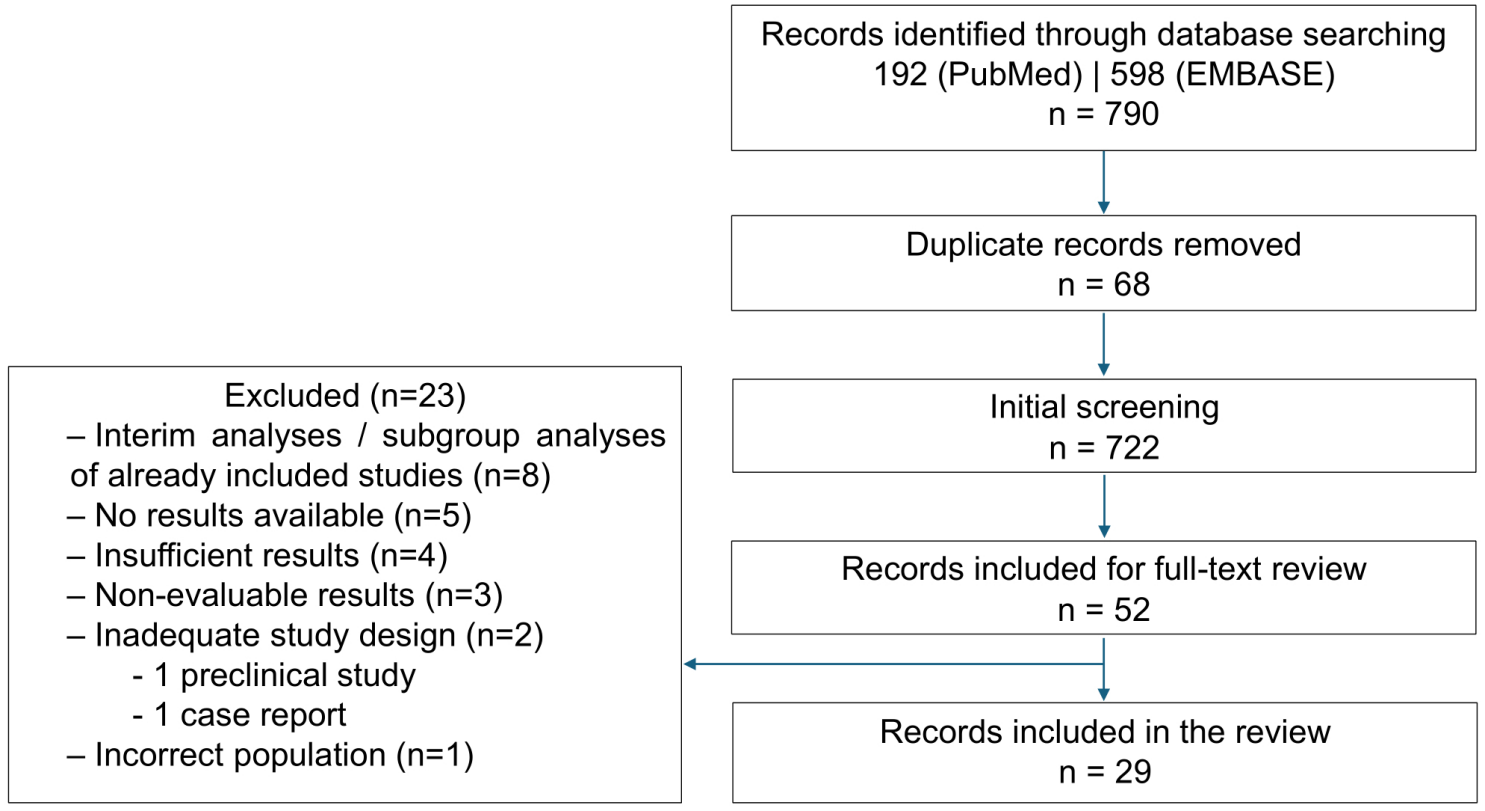
Study flowchart.

Thus, a total of 29 studies were included in this systematic review (**Figure 2**). All included studies were single-arm phase I/II trials, except Lee et al. (13), which was a randomized phase II trial.

**Tables 1**–**4** show the characteristics of the included studies.

**Table 1:** Use of NK adoptive transfer therapy in relapsed/refractory AML.

Study	Age range (median)	N	Intervention	Response (%)	HSCT after NK (%)	DFS (%)	OS (%)	GVHD (%)
Ahmadvand, et al (2023) (14)	50.5 (29-61)	10	2 x NK	0	0	1y:0	1y: 0	0
Ciurea et al (2023) (15)	60 (25-70)	12	6 x NK	58	42	1y: 25	1y:42	0
Pfeiffer et al (2024) (16) and Bednarski et al (2022) (17)	8	18	1 x ML-NK^a^	44	6	NR	1y:37	6
Silla et al (2021) (18)	22 (1.6-61)	13^b^	6 x NK	62^c^	39	NR	1y:27	8
Cooley et al (2019) (19)	52 (22-68) IV 63 (20-71) SC^d^	42	1 x NK + IL-15 IV or SC	35^e^	28 ^e^	1y: 12-19 ^e^	1y: 19-21 ^e^	0
Bjorklund et al (2018) (20)	64 (40-70)	16^f^	1 x NK	38^g^	31	1y:33	1y:44	NR
Boyiadzis et al (2017) (21)	71 (56-80)	7	2 x NK	0	0	1y:0	NR	NR
Romee et al (2016) (22, 23)	72 (43-83)	15^h^	1 x ML-NK + IL-2 SC	67	7	1y:10	1y:20	0
Shaffer et al (2016) (24)	19 (2-56)	8^i^	1 x NK + IL-2	16^j^	NR	0	NR	0
Bachanova et al (2014) (25)	Cohort 1:46 (7-68); Cohort 2:37(5-65); Cohort 3:51 (3-71)^k^	57	1 x NK + IL-2	30	18	Cohort 1 and 2: 5 Cohort 3: 33	NR	0
Miller et al (2005) (26)	NR	19^l^	1 x NK +IL-2 SC	26	NR	NR	NR	0

The intervention refers to the number of NK cell doses administered and whether their use was associated with systemic interleukins. The dose is presented as cells per kilogram of patient body weight per dose × 10^6^, except for one study that reported it as cells per m² of body surface area per dose. GVHD was assessed based on the proportion of patients who developed GVHD after NK cell therapy. All numbers greater than or equal to 1 were rounded to have no decimal places. N, number of patients treated with NK cells; HSCT, hematopoietic stem cell transplantation; DFS, disease-free survival; OS, overall survival; GVHD, graft-versus-host disease; NK, natural killer cell; ML-NK, memory like- natural killer cell; IL, interleukin; SC, subcutaneous; IV, intravenous; FluCy, Fludarabine and Cyclophosphamide; FluAraC, Fludarabine and Cytarabine; TLI, total lymphoid irradiation; D, day; y, years; N/R, not reported.

a. Four patients received two doses of ML-NK.

b. A total of 13 patients were treated, including one patient who received two treatments. Response was assessed per patient, not per infusion.

c. The results were presented per treated patient (rather than per number of infusions) and partial responses were not considered.

d. Divides the group into two cohorts (SC vs IV IL-15). Age is represented by the median of each cohort and the age extremes.

e. Response was assessed in each cohort. Two patients were excluded from the analysis due to premature death (total of 40 evaluated).

f. Treated patients included 5 high-risk myelodysplastic syndromes (MDS), 8 MDS/AML, and 3 AML.

g. Result disregarding patients with partial response.

h. Includes 14 AML patients and 1 with MDS.

i. Includes 6 AML patients and 2 with MDS.

j. Only results from the 6 AML patients were considered.

k. Three cohorts (different NK selection methods; 1 cohort also received IL2DT). Age is represented by the median range and age extremes of each cohort.

l. Only results from the 19 AML patients were considered.

**Table 2:** NK adoptive transfer therapy in pediatric and young adult patients with low- or intermediate-risk AML in response post-consolidation.

Study	Age median (range)	N	Intervention	DFS (%)	OS (%)	GVHD (%)
Garcia, et al (2021) (27)	7 (0.8.-16)	7	2 x NK + IL-2	3y: 71	3y:83	0
Nguyen, et al (2019) (28)	3 (0.1-15)	21	1 x NK + IL-2	~4.5y^a^:61	~4.5y^a^:84	0
Rubnitz, et al (2010) (29)	2.5 (0.2-21)	10	1 x NK + IL-2	2y: 100	2y:100	0

The intervention refers to the number of NK cell doses administered and whether their use was associated with systemic interleukins. The dose is presented as cells per kilogram of patient body weight per dose × 10^6^. GVHD was assessed based on the proportion of patients who developed GVHD after NK cell therapy. All numbers greater than or equal to 1 were rounded to have no decimal places. N, number of patients treated with NK cells; DFS, disease-free survival; OS, overall survival; GVHD, graft-versus-host disease; NK, natural killer cell; IL, interleukin; FluCy, Fludarabine and Cyclophosphamide; D, day; y, years.

a. Median follow up of 1698 days.

**Table 3:** NK adoptive transfer therapy in high-risk AML patients ineligible for HSCT in response post-consolidation.

Study	Age median (range)	N	Intervention	DFS (%)	OS (%)	GVHD (%)
Fehniger, et al (2018) (30)	73 (57-79)	12	1 x NK	1y: 33 2y: 25	1y:42 2y: 25	0
Dolstra, et al (2017) (31)	72 (68-76)	10	1x NK	1y: 60	1y: 80	0
Curti, et al (2016) (32)	64 (53-73)	17	1x NK + IL-2	~2y^a^:56	NR	0
Kottaridis, et al (2015) (33)	67 (51-73)	7	1 x NK^b^	1y: 14	1y: 86 2y: 14	0
Curti, et al (2011) (34)	62 (53-73)	13^c^	1 x NK + IL-2	1y: 33^d^	NR	0

The intervention refers to the number of NK cell doses administered and whether their use was associated with systemic interleukins. The dose is presented as cells per kilogram of patient body weight per dose × 10^6^. GVHD was assessed based on the proportion of patients who developed GVHD after NK cell therapy. All numbers greater than or equal to 1 were rounded to have no decimal places.

N, number of patients treated with NK cells; DFS, disease-free survival; OS, overall survival; GVHD, graft-versus-host disease; NK, natural killer cell; IL, interleukin; FluCy, Fludarabine and Cyclophosphamide; FluTBI, Fludarabine and TBI, Total Body Irradiation; D, day; SC, subcutaneous; y, years; N/R, not reported.

a. Median follow up of 22.5 months (range, 6–68 months).

b. One patient received a second dose of NK cell.

c. 13 patients, including 5 refractory, 2 relapsed, and 6 in complete remission. One patient received a second dose of NK cell.

d. Four patients with active disease did not respond. DFS was calculated for the nine patients who were either already in response at the time of infusion or achieved a response afterward.

**Table 4:** NK adoptive transfer therapy in AML patients undergoing HSCT.

Study	Age median (range)	N	Intervention	DFS (%)	OS (%)	aGVHD (%)	cGVHD (%)
Naik, et al (2024) (35)	8 (0.6-21)	72^a^	Haplo HSCT + 1 x NK	1y: 66^b^ 3y: 57^b^	1y: 79^b^ 3y: 67^b^	G3-4: 34^b^	mod-sev 1y: 13^b^
Lee, et al (2023) (36)	56 (21-70)	40^c^	Haplo HSCT + 2 x NK	2.5y: 33	2.5y: 35	Total:56 G3-4:35	Total:33 mod-sev: 20
Ciurea, et al (2021) (37)	46 (18-60)	24^d^	Haplo HSCT + 3 x NK	1y: 71 2y: 66	1y: 75 2y: 70	Total:42 G3-4: 4	Total:0 mod-sev: 0
Lee, et al (2016) (38)	51 (2-63)	21^e^	10/10 HLA- matched HSCT + 1xNK +/- IL-2^f^	1y: 28^g^	1y: 24	Total:33 G3-4:10	Total:29 mod-sev: 24
Choi, et al (2016) (39)	39 (19-67)	51^h^	Haplo HSCT + 4 x NK	3y: 9	3y: 21	Total:34 G3-4:22	Total:30 mod-sev: 28
Choi, et al (2014) (40)	47 (17-75)	41^i^	Haplo HSCT + 2 x NK^j^	~2.5y:31^k^	~2.5y:35^k^	Total:22 G3-4:12	Total:24 mod-sev: 15
Killig, et al (2014) (41)	43 (19-54)	24	Haplo HSCT + 1 x NK	NR	2y: 37	Total:83 G3-4:29	Total:0
Stern, et al (2012) (42)	(8-32)	16^l^	Haplo HSCT + 2-3 x NK^m^	NR	1y: 44 2y: 25 5y: 25	Total:25 G3-4:13	NR
Yoon, et al (2010) (43)	40 (23-65)	14^n^	Haplo HSCT + 1 x NK	1y: 50^o^	1y:64	Total:7 G3-4:0	Total:36 mod-sev:18^p^

The intervention refers to the number of NK cell doses administered and whether their use was associated with systemic interleukins. The dose is presented as cells per kilogram of patient body weight per dose × 10^6^. GVHD was assessed based on the proportion of patients who developed GVHD after NK cell therapy. All numbers greater than or equal to 1 were rounded to have no decimal places.

N, number of patients treated with NK cells; DFS, disease-free survival; OS, overall survival; aGVHD, acute graft-versus-host disease; cGVHD, chronic graft-versus-host disease; haplo, haploidentical; HSCT, hematopoietic stem cell transplantation; NK, natural killer cell; IL, interleukin; TLI, total lymphoid irradiation; FluCy, Fludarabine and Cyclophosphamide; Thio, thiotepa; Mel, melphalan; HPC, hematopoietic progenitor cells; BuFlu, Busulfan and Fludarabine; FluMelTBI, Fludarabine, melphalan and TBI, Total Body Irradiation; FluTBI, Fludarabine and TBI; D, day; y, years; G3-4, grade 3 to 4; mod-sev, moderate to severe; N/R, not reported .

a. The cohort included 72 patients: 38 with AML, 1 with MDS, 1 with myeloid sarcoma, 24 with B-Acute Lymphoid Leukemia (ALL), 5 with T-ALL, 2 with biphenotypic leukemia, and 1 with Non Hodgkin Lymphoma (NHL). Only 62 patients received NK cell infusion.

b. These results refer only to AML patients.

c. This was a randomized study. The total cohort included 76 patients (71 AML and 5 MDS): 40 were assigned to receive HSCT with NK cell infusion (38 AML and 2 MDS), and 36 to HSCT without NK cell infusion. Among the 40 patients in the NK group, 4 did not receive the infusion, and 4 received only a single dose. Only data from the cohort randomized to receive NK cell infusion are presented.

d. The cohort included 24 patients: 13 AML, 7 Chronic Myeloid Leukemia (CML) and 4 MDS.

e. The cohort included 21 patients: 8 AML, 7 CML and 6 MDS.

f. Ten patients received daily subcutaneous IL-2 for five days.

g. DFS was calculated for the 18 patients who were either already in response at the time of infusion or achieved a response afterward.

h. The cohort included 51 patients: 45 AML and 6 ALL.

i. The cohort included 41 patients: 32 AML, 7 ALL, 1 MDS and 1 NHL.

j. Four patients did not receive the second infusion of NK.

k. Results refer to AML patients. Median follow-up was 31.5 months (range, 16.6–53.0 months).

l. The cohort included 16 patients: 8 AML, 5 ALL, 2 Hodgkin Lymphoma (HL) and 1 Sarcoma.

m. Five patients received a single NK cell infusion, nine received two infusions, and two patients received three.

n. The cohort included 14 patients: 11 AML, 2 MDS and 1 ALL.

o. DFS was calculated for the 12 patients who were either already in response at the time of infusion or achieved a response afterward.

p. Chronic GVHD was assessed in the 11 patients with available data.

### 3.2 Relapsed or refractory AML

Eleven studies (217 patients) were included, mainly in adult patients (14–26). Lymphodepletion regimens varied and included fludarabine plus cyclophosphamide, fludarabine plus cytarabine, or no chemotherapy. Prior chemotherapy exposure was inconsistently reported. Although the enrolled cohorts were characterized as heavily pretreated, only four trials explicitly described prior exposure to cytarabine-based intensive regimens; exposure to fludarabine was not reported outside its use as part of lymphodepleting conditioning.

All studies used allogeneic NK cells, predominantly from haploidentical donors, with a minority using unrelated donors, non-HLA-selected (**Supplementary Table 2**). Complete remission outcomes were reported either as CR alone or as composite endpoints including CRi (complete remission with incomplete hematologic recovery) and/or CRp (complete remission with incomplete platelet recovery).

**Figure 3:**
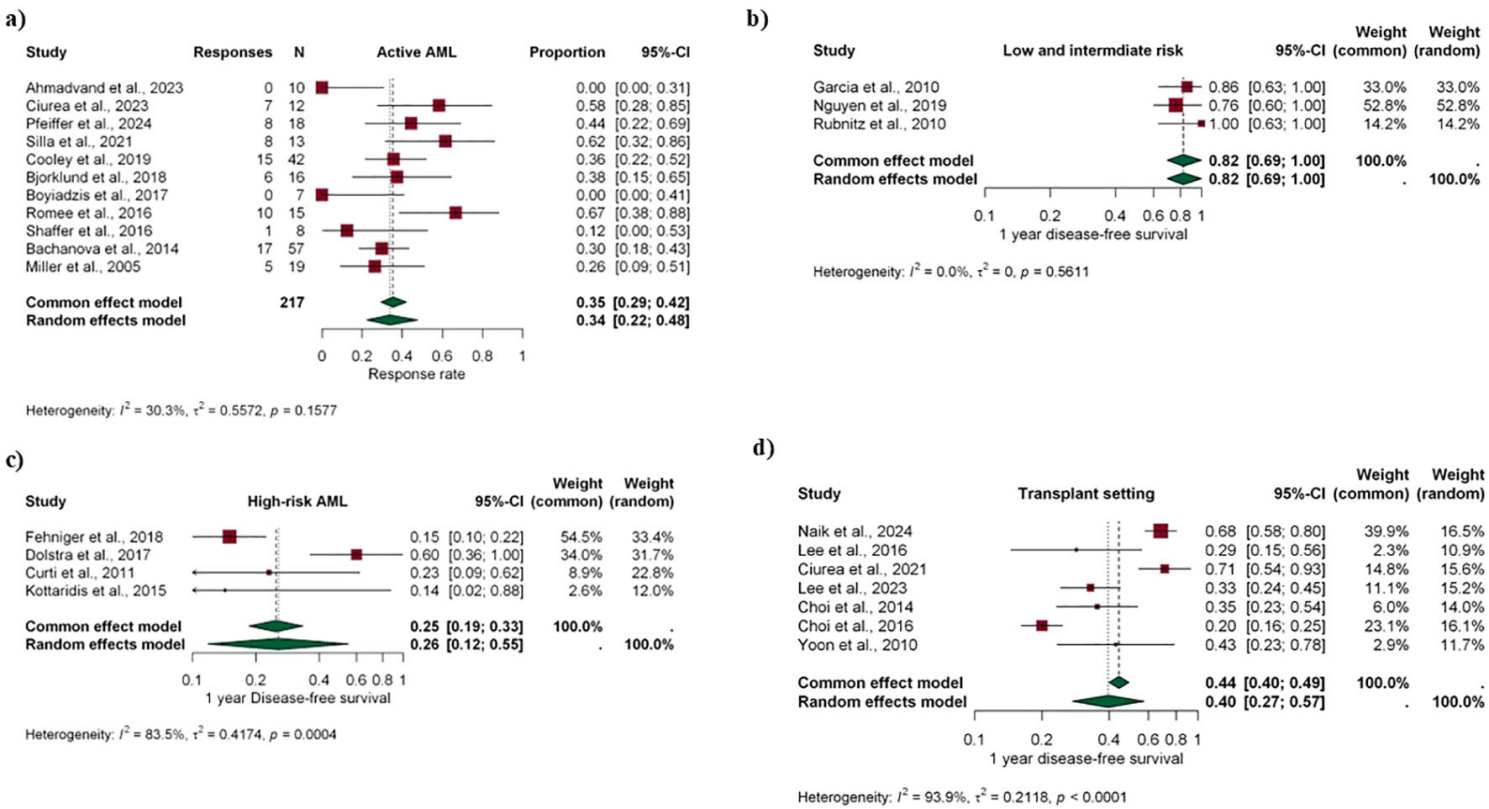
Forest plots of the primary outcome across different clinical scenarios. **(a)** shows the pooled complete response rate in patients with relapsed/refractory (R/R) AML. **(b–d)** present pooled 1-year disease-free survival according to risk category and transplant setting: **(b)** low- and intermediate-risk AML, **(c)** high-risk AML, and **(d)** transplant setting. Common- and random-effects models are shown, with corresponding 95% confidence intervals and heterogeneity estimates.

Pooled response rate was 35% (fixed-effect model, 95% CI 29–42%; **Figure 3a**). Despite the studies employing very different protocols, heterogeneity for response rate was relatively low (I² = 30%). We found no evidence of publication bias in the funnel plot analysis (p = 0.54, **Supplementary Figure 1a**). Overall, 20.2% (95% CI, 14.5–25.9) of patients received NK cell adoptive transfer as a bridge to HSCT. Among patients who achieved a clinical response, the depth of response, as assessed by measurable residual disease (MRD), was not systematically evaluated in most studies.

Across studies, higher response rates were more frequently observed in protocols designed to enhance NK cell activation and *in vivo* persistence, particularly those delivering six infusions of expanded NK cells supported by feeder cells or employing cytokine-induced memory-like NK cells (CIML-NK), with reported responses ranging from 44% to 67% (15–18, 22, 23). Notably, the trial using the NK-92 cell line without a lymphodepletion regimen failed to demonstrate any clinical benefit (21).

NK cell adoptive transfer was generally well tolerated, with low rates of GVHD (0–8%) and predominantly mild infusion-related reactions, such as fever, chills, and nausea. Silla et al. (18) reported one case of grade 4 neurotoxicity. Cooley et al. (19) reported nine cases of cytokine release syndrome (4 grade ≥3) and five cases of neurotoxicity, all in the subcutaneous IL-15 cohort. Severe toxicity was uncommon.

### 3.3 Low- or intermediate-risk AML in remission

Pediatric and young adult patients with low- or intermediate-risk AML in clinical remission after initial treatment were included, totaling 38 patients (27–29). Lymphodepletion consisted of fludarabine and cyclophosphamide. All studies assessed KIR mismatch, with the proportion of NK cell infusions involving a mismatch ranging from 57% to 100% (**Supplementary Table 3**). Pooled 1-y DFS was 82% (95% CI 56-100), with 0% heterogeneity (**Figure 3b**).

### 3.4 High-risk AML in remission and ineligible for HSCT

59 patients received a single NK cell infusion, with or without IL-2 support (30–34).

Prior therapies were inconsistently reported. Among studies with available data, most had achieved remission after intensive induction chemotherapy, typically anthracycline- and cytarabine-based regimens. A smaller subset entered remission following HMA-based therapy. There are no reports of prior exposure to venetoclax plus HMA.

Lymphodepletion included fludarabine plus cyclophosphamide and fludarabine combined with total body irradiation (TBI). Peripheral blood was the NK cell source in four studies, whereas one study used umbilical cord blood–derived NK cells. All donors were allogeneic (**Supplementary Table 4**).

Pooled 1y-DFS was 26% (95% CI 12-55), with high heterogeneity (84%) (**Figure 3c**). Similarly, no NK-related GVHD or major infusion-related events were reported. The FluTBI regimen was associated with severe and prolonged cytopenias, requiring additional CD34+ cell support.

### 3.5 NK cell adoptive transfer in association with HCT

Nine studies (303 patients) investigated the use of NK cell adoptive transfer in association with haploidentical HSCT, often in patients with AML (72%) (13, 35–42). The number of NK cell doses ranged from 1 to 4, and one study also included subcutaneous IL-2 administration.

Conditioning regimens varied widely. Across all studies, peripheral blood served as the NK cell source, and donors were consistently haploidentical. For stem cell donation, most studies used the same haploidentical donor for HSCT combined with NK cell infusion, whereas only one study employed a 10/10 HLA-matched donor. KIR mismatch was assessed in 89% of studies, with the proportion of infused alloreactive NK cells ranging from 29% to 81% (**Supplementary Table 5**).

The infusion timing ranges from day −8 (before HCT) to day +100.

Pooled 1y-DFS was 40% (95% CI 27-57), with a 94% heterogeneity (**Figure 3d**).

### 3.6 NK cell source, manufacturing, and dosing

NK cell intervention strategies were heterogeneous in terms of cell source, manufacturing and dosing. All products in the R/R AML setting were allogeneic, predominantly derived from haploidentical donors, except for non-HLA selected cryopreserved NK-92 cells (21) and a single study that employed unrelated healthy donors (14). In addition, KIR mismatch was evaluated in most studies, but the results varied considerably. Most studies used feeder-based expansion and cytokine-driven platforms. However, NK cell yields, the concomitant administration of systemic IL-2 and IL-15, the use of CIML-NK cell, and dosing strategies remained highly heterogeneous, these different approaches included single or multiple infusions, with cumulative doses ranging from 1 to 26 x10^6^ cells/kg. (**Supplementary Table 2**). 

Detailed information on NK cell source, manufacturing, and dosing for the remaining scenarios is provided in **Supplementary Tables S2**-**S5**.

**Figure 4:**
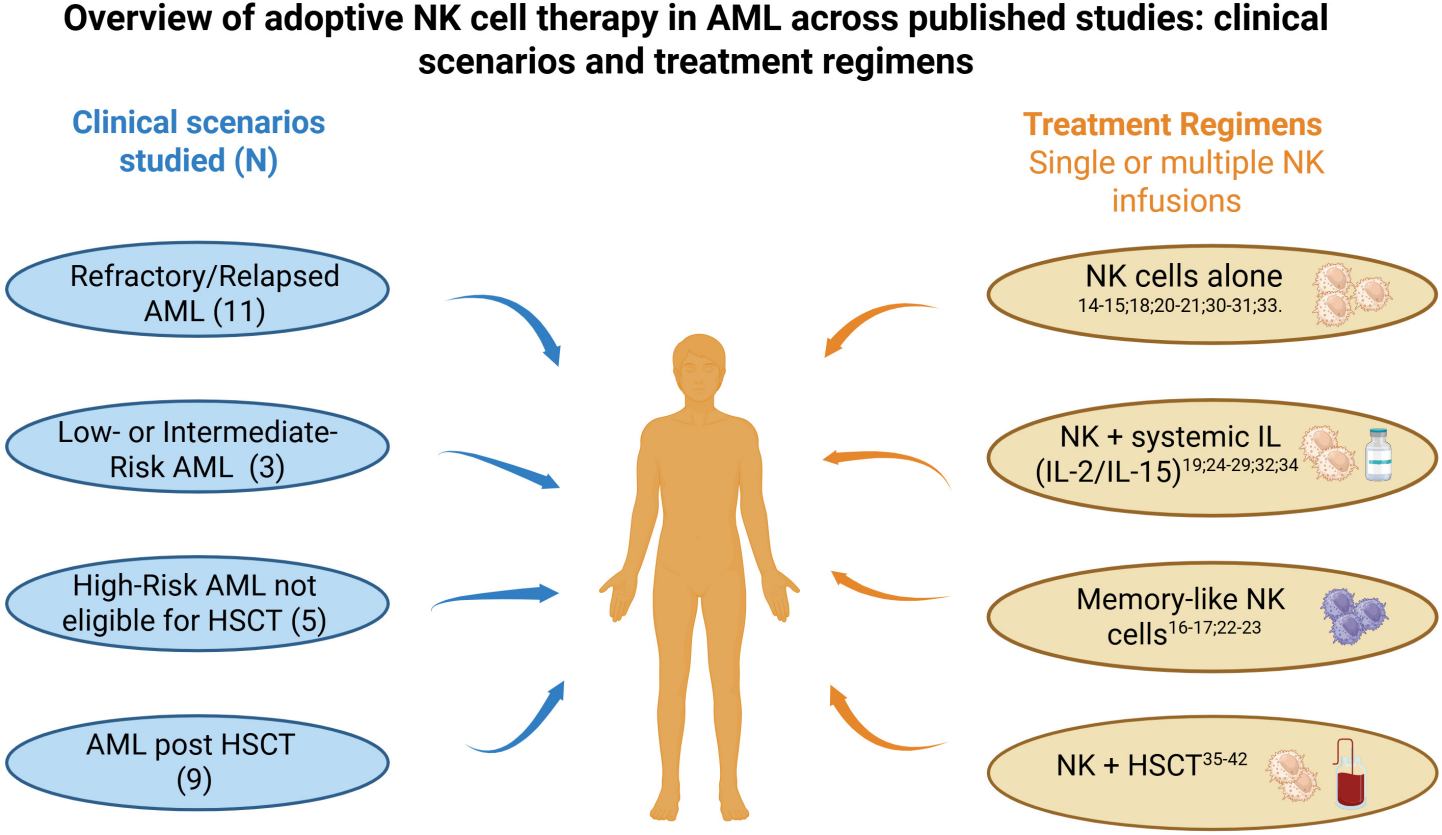
Adoptive NK cell therapy in AML: clinical settings and treatment regimens. This figure integrates clinical scenarios and treatment regimens reported across early-phase studies of adoptive NK cell therapy in acute myeloid leukemia. AML, Acute myeloid leukemia; N, number of studies; HSCT, hematopoietic stem cell transplantation; IL, interleukin.

An overview of the clinical scenarios and treatment regimens used across studies is summarized in **Figure 4**.

### 3.7 NK cell persistence

In R/R AML, NK cell persistence was generally short-lived, with NK cell quantification most commonly reported between days 7 and 14 post-infusion. Studies employing multiple infusions demonstrated that NK cells remained detectable for approximately 3 to 4 weeks (15, 18). Whereas CIML-NK cells studies described longer persistence, ranging from approximately 3 weeks to 3 months (16, 17, 22, 23). In low-risk AML and high-risk patients ineligible for HSCT, despite heterogeneous reporting, NK cell persistence remained consistently low, peaking between days 7 and 14 and followed by an early decline.

### 3.8 Quality assessment

Quality evaluation of the studies is available in the **Supplementary Material** (**Supplementary Table 1**). The funnel plots are also available in the **Supplementary Material** (**Supplementary Figure 1**).

## 4 Discussion

This systematic review of early-phase clinical trials shows the feasibility and low toxicity of NK cell adoptive transfer for AML. Response rates as sole therapy in relapsed patients remain lower than expected for a paradigm shift, and studies in other settings do not allow assessment of its true efficacy.

Although response rates as sole therapy in relapsed and refractory patients were not as high, the results in this scenario are encouraging, given the dismal prognosis of this population (43). A subset of responding patients was even able to undergo hematopoietic cell transplantation, currently the only curative option in this setting (**Table 1**). Studies evaluating venetoclax plus HMA in the relapsed/refractory AML setting have reported CR/CRi rates between 35% and 59% (44), quite similar to NK cell adoptive transfer in our study, and results comparable to intensive chemotherapy in other studies (45). These results suggest that,although NK cell adoptive transfer has shown encouraging activity in R/R AML, its use should remain restricted to prospective clinical trials, given the early-phase nature of the available evidence and the absence of definitive efficacy data (44, 45). Targeted therapies may offer the highest responses. In FLT3-mutated R/R AML, the response rate with gilteritinib was 54%, compared with 22% with conventional chemotherapy (46). Other targeted therapies include IDH1 inhibitors (from 30% to 41% response rates) (47, 48), and menin inhibitors (30% response rate) (49). Unfortunately, targeted therapies will be available only to a subset with a specific molecular profile. On the other hand, NK cell adoptive transfer could be offered to every patient with R/R AML in the future.

NK cell adoptive transfer has been evaluated as adjuvant therapy in only three studies in low- to intermediate-risk AML patients in remission. Not only were 3 studies included in our review, but the follow-up was mainly short. Although results seemed good, this was probably related to the patients’ disease profile. This, combined with the lack of a control group, hampers the analysis of efficacy. For this group, NK cell adoptive transfer should be only offered in clinical trials.

Disease-free survival in patients with high-risk AML ineligible for HSCT who received NK cell adoptive transfer was low. It was a predominantly older, and therefore, higher-risk population, largely unexposed to venetoclax, as these studies were conducted before widespread adoption as standard of care in this population (50, 51), and had heterogeneous disease status. The efficacy of NK-cells in high-risk AML patients ineligible for HSCT could not be isolated. If future studies demonstrate clinical benefit, NK cell adoptive transfer can even be combined with venetoclax to improve outcomes in this high-risk population. R/R AML, whether transplant-eligible or ineligible, remains a relevant unmet medical need, and studies with NK cell adoptive transfer represents one of the most appealing strategies (52).

We also included studies of NK cell adoptive transfer in haploidentical HSCT. Results were quite heterogeneous, mainly due to differences in disease proportions and in the proportions of relapsed/refractory disease. However, severe forms of graft-versus-host disease were not higher than expected. The main objective of these studies, i.e., to reduce relapse rates, could not be assessed (53–55).

Biological heterogeneity across studies limited meaningful disease and age-based stratification. Within this complex landscape, no clear association between KIR mismatch and response rates was observed, likely due to multiple uncontrolled confounders (34). In contrast, CIML-NK cells, generated through brief priming with IL-12, IL-15, and IL-18, undergo stable epigenetic and transcriptional reprogramming that enhances effector gene accessibility, metabolic fitness, and responsiveness to IL-15, collectively supporting improved *in vivo* persistence (22, 56, 57). Recent studies have therefore focused on CIML-NK cell-based strategies, including combination therapies with immune checkpoint inhibitors in solid tumors (58, 59) as well as an ongoing early-phase trial for AML (NCT07011004). In the post-haploidentical transplantation setting, a phase I study demonstrated the safety and feasibility of CIML-NK cell infusion (60), providing the rationale for an ongoing phase II trial (NCT02782546).

The studies did not detect any serious toxicity concerns. This is an important finding, since most studies were in phase I, where the focus is not on efficacy but on safety. It means phase II can proceed and start testing for efficacy outcomes. On the other hand, the number of infusions, the cell dose, the use of feeder cells or interleukin, the cellular platform, the NK source, the KIR mismatch, and the conditioning regimen varied widely across the studies, and these factors may have influenced the results. Limited and short-lived *in vivo* persistence of infused NK cells was a consistent finding across studies. Memory-like NK cells were associated with longer persistence in circulation, whereas protocols employing multiple infusions appeared to increase NK cell exposure. Whether a higher frequency of NK cell infusions translates into improved NK cell persistence remains an open question.

Our study has several limitations. The studies included were small and heterogeneous, as expected in phase I trials with an incipient technology. NK cell production was not uniform across protocols and techniques. Even the definition of response was not uniform across studies. Cytogenetic and molecular risk factors, as well as prior lines of therapy, were not always reported. On the other hand, 217 patients were included in this analysis. This systematic review is the most extensive conducted on allogeneic NK cell adoptive transfer.

The rapidly evolving therapeutic landscape of AML underscores the need to contextualize NK cell–based therapies within current standards of care. Given the established role of venetoclax combined with HMA have become a reference regimen for patients ineligible for intensive chemotherapy, with demonstrated survival benefits in both frontline and relapsed/refractory settings (50, 51), and has more recently been extended to newly diagnosed, fit patients eligible for intensive chemotherapy (61). Notably, none of the studies included in this review reported prior venetoclax exposure, precluding any assessment of potential priming or resistance effects. Nevertheless, the established efficacy of venetoclax-based regimens as a bridge to transplantation supports their consideration as clinically meaningful backbone therapies for future NK cell trials. In addition, venetoclax combined with an HMA should be considered a clinically meaningful low-intensity backbone comparator, particularly in unfit patients with relapsed/refractory AML, with stratification by prior venetoclax exposure and disease setting.

In conclusion, adoptive allogeneic NK cell transfer appears to be a safe and promising strategy and may be a valuable option in the future, especially for patients unfit for intensive chemotherapy or HCT. Currently, it should be restricted to clinical trials. Future studies should identify the optimal platform that balances clinical activity, low toxicity, and long-term persistence of allogeneic NK cells.
