# Supplementary material for: NK cell adoptive transfer in acute myeloid leukemia: a systematic review and meta-analysis
Source: Front Immunol. 2026 Mar 25;17:1717413. doi: 10.3389/fimmu.2026.1717413 (PMC13057487; doi:10.3389/fimmu.2026.1717413)
Supplement: Supplementary file 1 [file DataSheet1.pdf]

Supplement material

1. Search Methods

For natural killer cells, we used the following Medical Subject Headings (MeSH): “natural killer cell,” “NK cell,” “natural killer cells,” or “NK cells.” For acute myeloid leukemia, we used the descriptor “leukemia.” The search was intentionally broad, allowing descriptors to appear in any part of the manuscript to maximize sensitivity.

In PubMed, the search string was: ("natural killer cell" OR "NK cell" OR "NK Cells" OR "natural killer") AND ("Leukemia"), with filters for “clinical trial” and “randomized controlled trial” applied for the specified period.

In EMBASE, the search strategy was: #1: 'natural killer cell' AND 'leukemia'; #2: #1 AND ('clinical trial'/de OR 'randomized controlled trial'/de) AND [2000–2024]/py.

2. Figures

Figure S1. Funnel Plot for Assessment of Publication Bias across different clinical scenarios

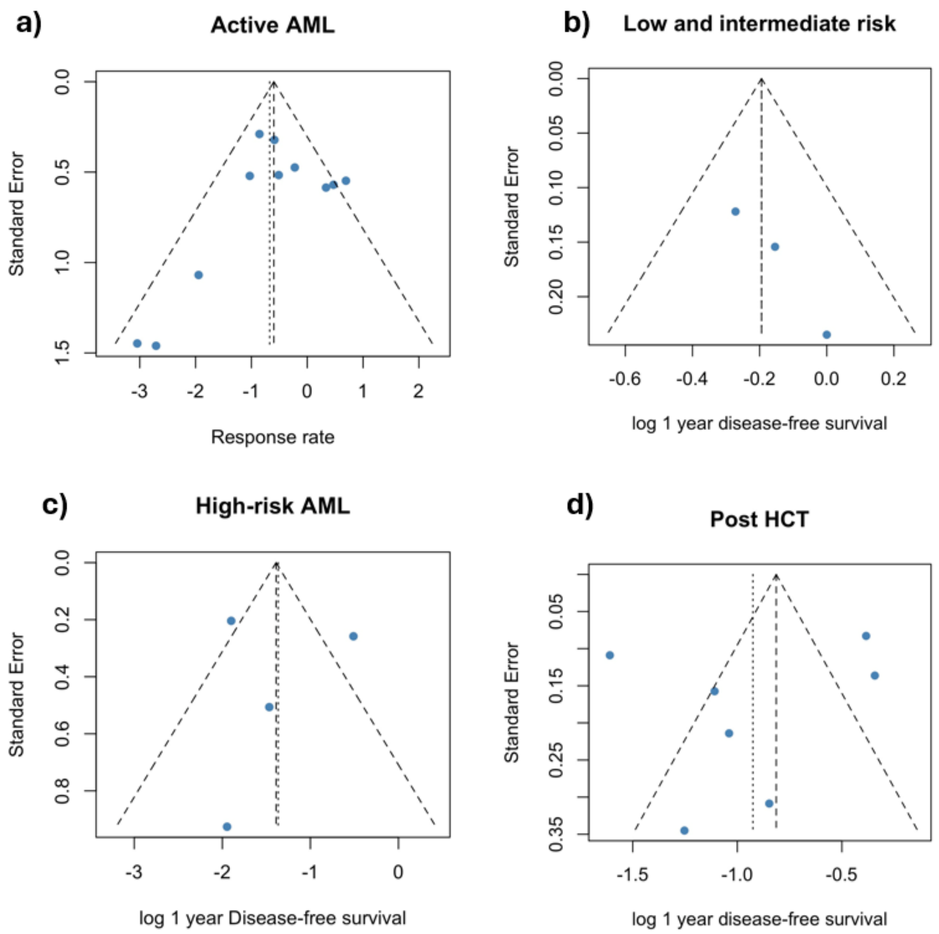

Legend: The panels show publication bias in patients with (a) relapsed/refractory (R/R) AML, (b) low- and intermediate-risk AML, (c) high-risk AML, and (d) the transplant setting.

3. Tables

Table S1. Quality assessment

Study	Global assessment	Study	Global assessment	Study	Global assessment
**Ahmadvan, et al (2023)**[15]	Low	**Miller, et al (2005)**[27]	Low	**Lee, et al (2023)**[37]	Moderate
**Ciurea, et al (2023)**[16]	Moderate	**Garcia, et al (2021)**[28]	Low	**Ciurea, et al (2021)**[38]	Moderate
**Pfeiffer, et al (2024)**[17] **and Bednarski, et al (2022)**[18]	Moderate	**Nguyen, et al (2019)**[29]	Moderate	**Lee, et al (2016)**[39]	Moderate
**Silla, et al (2021)**[19]	Moderate	**Rubnitz, et al (2010)**[30]	Moderate	**Choi, et al (2016)**[40]	Moderate
**Cooley, et al (2019)**[20]	Moderate	**Fehniger, et al (2018)**[31]	Moderate	**Choi, et al (2014)**[41]	Moderate
**Bjorklund, et al (2018)**[21]	Moderate	**Dolstra, et al (2017)**[32]	Moderate	**Killig, et al (2014)**[42]	Low
**Boyiadzis, et al (2017)**[22]	Moderate	**Curti, et al (2016)**[33]	Moderate	**Stern, et al (2012)**[43]	Low
**Romee, et al (2016)**[23,24]	Low	**Kottaridis, et al (2015)**[34]	Low	**Yoon, et al (2010)**[44]	Moderate
**Shaffer, et al (2016)**[25]	Low	**Curti, et al (2011)**[35]	Moderate		
**Bachanova, et al (2014)**[26]	Moderate	**Naik, et al (2024)**[36]	Moderate		

Legend: Studies were classified in the global assessment as being of low, moderate, or high quality.

Table S2. Sources, Manufacturing Methods, and KIR Mismatch in NK Cell Therapy for R/R AML

Study	Source	Donor	Manufacturing	KIR mismatch (%)	Dose $x10^6$ cells/kg range (median)	Treatment regimen
**Ahmadvand, et al (2023)[14]**	PB (WB)	URD	CD3 depletion; AB serum, IL-2, autologous PBMNC and anti-CD3 for 21 days	90	1st dose:2-5<br>2nd dose:5-10	FluCy;<br>NK on D0 and D+7
**Ciurea et al (2023)[15]**	PB (WB)	Haplo	CD3 depletion and weekly stimulation with FC21 for 14 days	42	1-10 (10)	FluAraC. NK on D0 and every other day
**Pfeiffer et al (2024)[16] and Bednarski et al (2022)[17]**	PB (Apheresis)	Allogeneic	CD3 depletion and CD56 selection; stimulation with IL-12, IL-15, IL-18	NR	4-10	FluAraC. DI on D-1. NK on D0
**Silla et al (2021)[18]**	PB (WB)	Haplo	CD3 depletion; stimulation with K562 cell feeder	46	1-10	FluAraC. NK on D0 and every other day
**Cooley et al (2019)[19]**	PB (Apheresis)	Haplo	CD3 and CD19 depletion; stimulation with IL-15 overnight	29	12-19[a]	FluCy; NK on D0. IL-15 IV or SC
**Bjorklund et al (2018)[20]**	PB (Apheresis)	Haplo	CD3 and CD19 depletion; stimulation with IL-2 overnight	48	1 -18 (7)	FluCy + TLI. NK on D0.
**Boyiadzis et al (2017)[21]**	NK92	URD	Expansion of NK92 cells using AB serum, IL-2, asparagine, L-glutamine, and L-serine	NR	$13x10^9$cells/m[2b]	No conditioning. Two doses of NK cells administered 24 hours apart.
**Romee et al (2016)[22,23]**	PB (Apheresis)	Allogeneic	CD3 depletion and CD56 selection, followed by 12-hour activation with IL-12, IL-15, and IL-18	NR	0.5-10	FluCy. NK on D0, followed by IL-2 SC every other day (6 doses)
**Shaffer et al (2016)[24]**	PB (Apheresis)	Haplo	CD3 depletion and CD56 selection	63	4-22 (11)	FluCy. NK on D0, followed by IL-2 every other day (6 doses)
**Bachanova et al (2014)[25]**	PB (Apheresis)	Haplo	CD3 depletion alone, combined with CD19 depletion, or combined with CD56 selection Activation with IL-2.	33	Cohort 1: 10<br>Cohort 2: 3<br>Cohort 3: 26	FluCy. NK on D0. IL-2 SC every other day (6 doses). (in one cohort ILD2T on D-1 ± D -2)
**Miller et al (2005)[26]**	PB (Apheresis)	Haplo	CD3 depletion; stimulation with IL-2 overnight	21	9 (2-15)	FluCy. NK on D0, followed by IL-2 every other day (6 doses)

Legend: Abbreviations: PB: peripheral blood; WB: whole blood; URD: unrelated donor; Haplo: haploidentical; NK: natural killer cell; PBMNC: peripheral blood mononuclear cells; IL: interleukin;N/R: not reported.

a. Data refers to the mean dose in each group.

b. Study in which the dose was calculated as cells per m² of body surface area per dose.

Table S3. Sources, Manufacturing Methods, and KIR Mismatch in NK Cell Therapy for Low- or Intermediate-Risk AML after Consolidation

Study	Source	Donor	Manufacturing	KIR Mismatch (%)	Dose $X10^6$ cells/kg Range (median)	Treatment regimen
**Garcia, et al (2021)[27]**	PB (WB)	Haplo	Selection with Ficoll-Paque; cultured with K562, IL-2, and AB serum for 3 weeks.	57	1st dose: 7-50 (24)<br>2nd dose: 17-193 (69)	FluCy. NK on D0 and D+7. IL-2 SC started on D-1, every other day (6 doses).
**Nguyen, et al (2019)[28]**	PB (Apheresis)	Haplo	CD3 depletion and CD56 selection.	100	4-62 (13)	FluCy. NK on D0. IL-2 SC started on D-1, every other day (6 doses).
**Rubnitz, et al (2010)[29]**	PB (Apheresis)	Haplo	CD3 depletion and CD56 selection.	90	5-81 (27)	FluCy. NK on D0. IL-2 SC started on D-1, every other day (6 doses).

Legend: Abbreviations PB: peripheral blood; WB: whole blood; haplo: Haploidentical; IL: interleukin.

Table S4. Sources, Manufacturing Methods, and KIR Mismatch in NK Cell Therapy for high-risk AML ineligible for HSCT

Study	Source	Donor	Manufacturing	KIR Mismatch (%)	Dose $X10^6$ cells/kg Range (median)	Treatment regimen
**Fehniger, et al (2018)[30]**	PB (Apheresis)	Haplo	CD56 selection, incubation with CNDO-109 lysate[a] for 16 hours	58	0.3-3 (2)	FluCy; NK on D0
**Dolstra, et al (2017)[31]**	Cord blood bank	Allogeneic	CD34 selection and cultured for 42 days with a cytokine cocktail.	70	3-30 (10)	FluCy; NK on D0
**Curti, et al (2016)[32]**	PB (Apheresis)	Haplo	NK-cell separation using CliniMACS; specific protocol not reported.	100	1 – 6 (4)	FluCy; NK on D0; IL-2 SC every other day (6 doses)
**Kottaridis, et al (2015)[33]**	PB (Apheresis)	Haplo	$CD56^+$ selection; overnight incubation with CTV-1[b] leukemia cell lysate	29	1	FluTBI; NK on D0
**Curti, et al (2011)[34]**	PB (Apheresis)	Haplo	CD3 depletion and CD56 selection	100	1-5 (3)	FluCy; NK on D0; IL-2 SC every other day (6 doses)

Legend: Abbreviations: PB: peripheral blood; Haplo: haploidentical.

a. CND0-109: a tumor cell–derived protein complex used as an NK cell–activating agent;
b. CTV-1: a T-cell leukemia cell line lysate used to activate (or prime) NK cells ex vivo.

Table S5. Sources, Manufacturing Methods, and KIR Mismatch in NK Cell Therapy for AML in conjunction with HSCT

Study	Source	Donor	Manufacturing	KIR Mismatch (%)	Dose $X10^6$ cells/kg Range (median)	Treatment regimen
**Naik, et al (2024)[35]**	PB (Apheresis)	Haplo (same as HSCT)	CD3 depletion followed by CD56 selection	62	1-99 (11)	TLI/FluCy/Thio/Mel; $CD34^+$-enriched HPC graft on D0; $CD45RA^-$ HPC graft on D0/+1; NK on D+6.
**Lee, et al (2023)[36]**	PB (Apheresis)	Haplo (same as HSCT)	CD3 depletion; stimulation with IL-15, IL-21 and hydrocortisone for 2-3 weeks	81	1st dose: 50-100 (100)<br>2nd dose: 50-400 (140)	BuFlu; HPC on D0; NK on D+13 and D+20
**Ciurea, et al (2021)[37]**	PB (WB)	Haplo (same as HSCT)	CD3 depletion; stimulation with FC21 for 14 days	NR	0.1-100	FluMelTBI; HPC on D0; Nk on D-2, D+7 and D+28
**Lee, et al (2016)[38]**	PB (Apheresis)	Haplo	CD3 depletion; stimulation with IL-2 overnight	76	0.02-8 (3)	BuFlu. NK on D-8. HPC on D0
**Choi, et al (2016)[39]**	PB (Apheresis)	Haplo (same as HSCT)	CD3 depletion; stimulation with IL-15, IL-21 and hydrocortisone for 2-3 weeks	26	1st and 2nd dose: 20-50 (50)<br>3rd dose: 70-210 (100)<br>4rh dose: 90-500 (200)	BuFlu. HPC on D0. NK on D+6,D+9,D+13 and D+20
**Choi, et al (2014)[40]**	PB (Apheresis)	Haplo (same as HSCT)	CD3 depletion; stimulation with IL-15, IL-21 and hydrocortisone for 2-3 weeks	17	1st dose: 20-200<br>2nd dose:20-500	BuFlu. HPC on D0. NK on 2nd and 3rd week
**Killig, et al (2014)[41]**	PB (Apheresis)	Haplo (same as HSCT)	CD3 depletion followed by CD56 selection	54	2-32 (8)	FluTBI, thiotepa; HPC on D0; NK on D+1
**Stern, et al (2012)[42]**	PB (Apheresis)	Haplo (same as HSCT)	CD3 depletion followed by CD56 selection	69	3-38 (12)	Conditioning regimen not specified.HPC on D0; NK on D+40 and D+100, with some patients also receiving a dose on D+3
**Yoon, et al (2010)[43]**	PB (Apheresis)	Haplo (same as HSCT)	$CD34^+$ selection; cultured with SCF, Flt3L, IL-7, and hydrocortisone for 21 days, followed by IL-15, IL-21, and hydrocortisone for 21days	29	0.3-24.5 (9.3)	BuFlu; HPC on D0 and D+1; NK at 6–7 weeks post-HPC.

Legend: Abbreviations: PB: peripheral blood; WB: whole blood; Haplo: haploidentical; ; IL: interleukin.
